# Overexpression of miR-451a Aggravates Renal Ischemia–Reperfusion Injury by Targeting KLF1-ACSL4 to Promote Ferroptosis

**DOI:** 10.3390/cimb46110704

**Published:** 2024-10-23

**Authors:** Haitao Yu, Xin Gou

**Affiliations:** 1 Department of Urology, The First Affiliated Hospital of Chongqing Medical University, Chongqing 400016, China; yuhaitao@stu.cqmu.edu.cn; 2 Chongqing Key Laboratory of Molecular Oncology and Epigenetics, The First Affiliated Hospital of Chongqing Medical University, Chongqing 400016, China

**Keywords:** ischemia–reperfusion injury, microRNA, ferroptosis, Kruppel-like factor 1, acyl-CoA synthetase long-chain family member 4

## Abstract

Ischemia–reperfusion injury (IRI) is a predominant factor leading to delayed graft function (DGF) following kidney transplantation. MicroRNAs (miRNAs) play a pivotal role in the pathogenesis of renal IRI, with ferroptosis being a critical driving force throughout the process. In this study, we utilized bioinformatics methods to construct a network diagram of differentially expressed miRNAs, transcription factors (TFs), and ferroptosis-related genes. An I/R-induced renal injury model in mice and an in vitro H/R-induced HK-2 cell injury model were established. Quantitative real-time PCR (qRT-PCR) and Western blot analysis were used to measure the mRNA and miRNA levels in cells and tissues. The MDA concentration, iron levels, and GSH concentration were measured to evaluate the ferroptosis levels. CCK-8 assays were performed to assess cell viability. Luciferase reporter assays were conducted to validate the downstream targets of miRNA, and chromatin immunoprecipitation assays were performed to verify the interaction between TFs and mRNAs. Both the in vivo and in vitro results demonstrate that miR-451a was significantly enriched in the IRI renal tissues and cells, exacerbating ferroptosis. MiR-451a was found to reduce the expression of Kruppel-like factor 1 (KLF1) by directly binding to the 3′UTR of KLF1 mRNA. Additionally, KLF1 was identified as a negative transcription factor for acyl-CoA synthetase long-chain family member 4 (ACSL4). We demonstrated that IRI induced the upregulation of miR-451a, which reduced KLF1 expression, thereby promoting ferroptosis by upregulating ACSL4 expression, ultimately aggravating IRI-induced renal damage.

## 1. Introduction

Ischemia–reperfusion injury (IRI) is characterized by cellular damage resulting from an inadequate oxygen supply to tissues, attributable to a compromised or complete cessation of blood flow to the affected area due to various etiologies. Ischemia–reperfusion typically exacerbates histopathological damage, thereby influencing disease progression and impeding the recovery process [1]. Kidney transplantation is currently a highly effective clinical treatment for end-stage renal disease [2]. However, during the acquisition and transplantation processes, kidneys are unavoidably subjected to IRI. This may result in delayed graft function (DGF) in the immediate post-transplant phase or, in more severe scenarios, total graft failure and potential allograft rejection [3]. Additionally, the significant disparity between organ supply and demand has led to the increased use of marginal donor kidneys, including those from donation after circulatory death (DCD) and extended criteria donors (ECDs), further exacerbating IRI-induced DGF [4]. Consequently, a deeper understanding of the mechanisms and patterns of IRI is crucial for the identification of novel therapeutic targets and precise biomarkers for prognostic evaluation.

Ferroptosis is a newly identified form of regulated cell death marked by iron-dependent lipid peroxidation [5]. Ferroptosis is primarily driven by the accumulation of lipid reactive oxygen species (ROS) due to the failure of glutathione-dependent antioxidant defenses, particularly the inactivation of glutathione peroxidase 4 (GPX4) [6]. Ferroptosis has been implicated in various pathological conditions, including neurodegenerative diseases [7], ischemia–reperfusion injury [8], and cancer [9]. Long-chain fatty acid-CoA ligase 4 (ACSL4) plays a central role in ferroptosis by positively regulating this process through the activation of long-chain fatty acid metabolism [10]. MicroRNAs (miRNAs) are a class of non-coding RNA transcripts, typically 20–22 nucleotides in length. miRNAs can bind to protein-coding messenger RNA (mRNA), leading to mRNA degradation or inhibition of translation, thus regulating various cellular processes, including metabolism and tumor progression [11]. During IRI, miRNAs are involved in the regulation of various cellular processes, including inflammation [12], apoptosis [13], oxidative stress [14], and autophagy [15]. Several miRNAs have been identified to modulate key signaling pathways related to IRI. For example, miR-21 is commonly upregulated during IRI and helps to reduce cell apoptosis and inflammation, thereby providing a protective effect [16]. Conversely, miR-485 [17] and miR-24 [18] are associated with promoting cell death and exacerbating damage during reperfusion. In addition, miR-20a-5p alleviates ischemia–reperfusion-induced acute kidney injury and postischemic fibrosis by targeting ACSL4 and inhibiting ferroptosis [19]. The interplay between ACSL4 and specific miRNAs may have significant implications for the regulation of ferroptosis and its associated pathophysiological outcomes.

Here, we present evidence that miR-451a is considerably upregulated in the IRI process and aggravates ferroptosis by reducing the KLF1-mediated suppression of ACSL4, thereby exacerbating tubular necrosis after renal transplant. Mechanically, miR-451a is highly enriched in IRI renal tubular epithelial cells, leading to the significantly reduced expression of KLF1, which was predicted and proven to be a transcription factor (TF) of ACSL4, provoking ferroptosis during the renal IRI process. Our findings suggest that miR-451a is a new biomarker and target for the early evaluation and treatment of IRI after renal transplant.

## 2. Methods

### 2.1. Data Collection

We utilized the R package GEOquery [20] to download gene and microRNA expression profiles from the GSE71647 and GSE172039 [21] datasets of renal IRI tissues. Transcription factor data were obtained from the TRRUST V2 database (https://www.grnpedia.org/trrust/, accessed on 12 October 2024). Ferroptosis-related genes were sourced from the FerrDb database (https://www.zhounan.org/ferrdb/current/, accessed on 12 October 2024).

### 2.2. Differentially Expressed Genes (DEGs)

Based on the grouping information within the data, we employed the R package limma [22] to analyze intergroup differences. Genes with a log fold change (logFC) > 0.5 and *p* < 0.05 were considered upregulated, while genes with logFC < −0.5 and *p* < 0.05 were considered downregulated. The R packages pheatmap and ggplot2 were used to visualize the differentially expressed genes (DEGs).

### 2.3. Construction of miRNA–TF–Ferroptosis Gene Network

We employed the miRNet database to identify differentially expressed miRNAs and transcription factors (TFs) associated with renal IRI. To visualize the miRNA–TF–ferroptosis gene network, we utilized Cytoscape software (Version 3.10.2).

### 2.4. Animals

Male C57BL/6 mice, aged 8–10 weeks, were utilized for the experiments. The mice were housed in a standardized facility at the Chongqing Medical University Animal Centre, maintained under constant temperature and humidity with a 12-h light/dark cycle, and regularly monitored by a certified veterinarian. All experimental protocols and animal care procedures complied with the Chongqing Medical University Policy on the Care and Use of Laboratory Animals (k276). The animals were randomly allocated using computer-generated numbers, and the investigators were blinded to group assignments during the data collection and analysis. This study followed the recommendations of the National Institutes of Health Guide for the Care and Use of Laboratory Animals. The protocol received approval from the Chongqing Medical University Committee on the Ethics of Animal Experiments (Protocol Number: k276). Surgeries were performed under sodium pentobarbital anesthesia, with all efforts made to minimize suffering.

### 2.5. In Situ Kidney IRI Model

To establish an in vivo model of in situ kidney warm ischemia–reperfusion, C57BL/6 mice were anesthetized and maintained on a thermostatic insulation pad. After performing a left nephrectomy, the right renal pedicle was occluded with a microvascular clamp to induce warm ischemia for 1 h, followed by clamp release for reperfusion. Sham-operated mice underwent the same procedure without pedicle clamping. The incision was then closed using 4-0 silk sutures. The mice were euthanized 1 day post-reperfusion to collect blood samples and the right kidney for analysis. Additionally, 24 h prior to surgery, the mice received tail vein injections of PBS, agomir NC, agomir, or antagomir.

### 2.6. In Vitro IRI Model

A chemical anoxia/recovery approach was utilized to establish an in vitro ischemia–reperfusion injury (IRI) model, as previously documented [23,24]. HK-2 cells were initially cultured in a glucose-free medium supplemented with 5 μM antimycin A and 5 mM 2-deoxyglucose to mimic the ischemic phase for 1 h. Subsequently, the cells were subjected to a reperfusion phase by replacing the medium with a complete medium for either 24 or 48 h.

### 2.7. Cell Culture and Treatment

The human proximal tubular cell line (HK-2), Normal Rat Kidney-52E (NRK-52E), and human embryonic kidney cell line (293T) were sourced from the Cell Bank of the Chinese Academy of Sciences (Shanghai, China). These cell lines were maintained in Dulbecco’s modified Eagle’s medium (DMEM)/F12 (Gibco, Waltham, MA, USA) with the addition of 10% fetal bovine serum (FBS, BioInd, Kibbutz Beit Haemek, Israel), 100 μg/mL streptomycin (Beyotime, Shanghai, China), and 100 U/mL penicillin (Beyotime, China) at 37 °C in a 5% CO_2_ humidified incubator. Specifically, the 293T cells were cultured in DMEM (Gibco, USA).

### 2.8. Oligonucleotides, Plasmids, and Cell Transfection

Full-length KLF1 cDNA was subcloned into the pcDNA3.1 vector. The miR-451a mimic, negative control mimic (mimic NC), and miR-451a inhibitor were synthesized by Tsingke Biotechnology. The sequences were as follows: hsa-miR-451a mimic (sense, 5′-AAACCGUUACCAUUACUGAGUU-3′; antisense, 5′-CACAAGUUCGGAUCUACGGGUU-3′), and hsa-miR-451a inhibitor (sense, 5′-AACUCAGUAAUGGUAACGGUUU-3′). The plasmids and oligonucleotides were transfected using Lipofectamine 3000 Transfection Reagent (Invitrogen, Waltham, MA, USA) according to the manufacturer’s instructions.

### 2.9. Quantitative Real-Time PCR (qRT-PCR)

Total RNA was extracted from the pretreated cells and mice tissues using TRIzol reagent (Abclonal, Wuhan, China). The purified RNA was then reverse-transcribed using the PrimeScript qRT-PCR kit (Abclonal, China). Subsequently, quantitative real-time PCR (qRT-PCR) was performed using the SYBR^®^ PrimeScript qRT-PCR kit (Abclonal, China) on an ABI 7500 Real-Time PCR System (Applied Biosystems, Waltham, MA, USA). The gene expression levels were evaluated using the 2^−ΔΔCt^ method. The primer sequences used for PCR were as follows: β-actin: forward primer: 5′-CCTCTGAACCCTAAGGCCAA-3′, reverse primer: 5′-GTCTCCGGAGTCCATCACAA-3′; Acsl4: forward primer: 5′-TGAACGTATCCCTGGACTAGG-3′, reverse primer: 5′-TCAGACAGTGTAAGGGGTGAA-3′; ACSL4: forward primer: 5′-TCTCTTGCCTCAGCCTCCTTAGTAG-3′, reverse primer: 5′-CGAGACCAGCCTGACCAACATG-3′; KLF1: forward primer: 5′-TTGCGGCAAGAGCTACACCAAG-3′, reverse primer: 5′-GTTGGTGACCAAGTGGCTGTAG-3′; Klf1: forward primer: 5′-AGACTGTCTTACCCTCCATCAG-3′, reverse primer: 5′-GGTCCTCCGATTTCAGACTCAC-3′.

### 2.10. Lipid Peroxidation, Iron, and Glutathione Assay

The malondialdehyde (MDA) levels were assessed using a lipid peroxidation assay kit (Abcam, Cambridge, UK). Briefly, standards and samples were incubated with TBA reagent at 95 °C for 1 h and then cooled to room temperature in an ice bath. Subsequently, 200 μL of the mixture was transferred to a 96-well plate, and the absorbance was recorded at 532 nm using a microplate reader (BioRad, Hercules, CA, USA).

Iron quantification was conducted with an iron assay kit (ElabScience, Wuhan, China). Homogenized samples in iron assay buffer underwent centrifugation at 16,000× *g* for 10 min at 4 °C. A 10 μL aliquot of the supernatant was combined with 90 μL of iron assay buffer, and then incubated with 5 μL of iron reducer at 25 °C for 30 min. Finally, the mixture was incubated with an iron probe in the dark, and the absorbance was measured at 532 nm.

The glutathione (GSH) levels were evaluated using a glutathione assay kit (Elabscience, CHN). To prevent GSH autoxidation and degradation, a 5% 5-sulfosalicylic acid solution was utilized. Following cell lysis, 10 μL of the supernatant was incubated with the reaction mix, and the GSH content was determined by measuring the absorbance at 405 nm.

### 2.11. Cell Counting Kit-8

Pre-prepared HK-2 cells were seeded on a 96-well plate at a density of 3 × 10^3^ cells per well, supplemented with 200 µL of DMEM/F12 medium containing 10% FBS. After incubation at 37 °C for 2 h, the absorbance was measured at 450 nm using a microplate reader.

### 2.12. Immunoblotting

Total protein samples were extracted from the cells and mouse renal tissues using radioimmunoprecipitation assay (RIPA) buffer (Beyotime, China) containing a protease inhibitor (Thermo Fisher Scientific, Waltham, MA, USA). Immunoblotting (IB) assays were then performed. The proteins were separated using sodium dodecyl sulfate–polyacrylamide gel electrophoresis (SDS-PAGE) and subsequently transferred onto polyvinylidene difluoride (PVDF) membranes (Millipore, Burlington, MA, USA). The protein blots were visualized using an enhanced chemiluminescent substrate (Bio-Rad, USA) and quantified using ImageJ software (version 1.54j 12) (NIH, Bethesda, MD, USA). The antibodies used in the study were as follows: ACSL4 (1:5000, Proteintech, San Diego, CA, USA, 22401-1-AP) and KLF1 (1:1000, ThermoFisher, PA5-86441).

### 2.13. Luciferase Reporter Assay

The 3′-UTR of KLF1, containing the miR-451a binding site, and a mutant 3′-UTR KLF1 sequence were cloned into the pmirGLO vector. Subsequently, 293T cells were co-transfected with these luciferase reporter constructs and either a miR-451a mimic or a negative control. The luciferase activity was measured using the Dual-Luciferase^®^ Reporter Assay System (Promega, Madison, WI, USA).

### 2.14. Chromatin Immunoprecipitation (ChIP) Assay

The ChIP assay, utilizing the EZ-ChIP Assay Kit (Millipore, MA, USA), was performed to explore the interaction between KLF1 and ACSL4. In brief, 2 × 10^6^ RCC cells were fixed with 1% formaldehyde to cross-link proteins to DNA. Following fixation, the cells were washed three times with PBS and lysed with a buffer containing PMSF and protease inhibitors. The lysate was then sonicated for 20–30 min to fragment the DNA. A 10% aliquot of the lysate was set aside as the input, while the remainder was incubated with anti-KLF1 antibody (ThermoFisher, PA5-86441) and anti-IgG antibody (Abcam, ab172730) at 4 °C for 16 h, followed by overnight incubation with A/G protein magnetic beads at 4 °C. The retrieved DNA was then quantified using qRT-PCR with the following primers: ACSL4: forward primer: 5′-CAGGCATGCTTACTGTCCAG-3′; reverse primer: 5′-TGAGCCTGCAAAACTTCCTG-3′.

### 2.15. Renal Morphology and Function Assessment

Hematoxylin and eosin (H&E) staining was employed to evaluate the renal histological morphology. Renal tubular injury was assessed based on the following criteria: tubular epithelial cell swelling, tubular atrophy and dilatation, loss of brush border, vacuolization, and cast formation. Renal function was evaluated by measuring the blood urea nitrogen (BUN) and serum creatinine (SCr) levels.

### 2.16. Immunohistochemistry (IHC)

The tissue was fixed in 4% buffered formalin (Biosharp, Beijing, China). Following dehydration, antigen retrieval, and blocking, the tissue was incubated overnight at 4 °C with primary antibodies. The membranes were subsequently incubated with secondary antibodies (Bioss, Beijing, China) for 1 h at room temperature. Sections were stained with diaminobenzidine (DAB, Sigma-Aldrich, Darmstadt, Germany) and counterstained with hematoxylin. The quantity of DAB-positive cells was assessed in 5 random fields using an optical microscope (Olympus, Tokyo, Japan). The following primary antibodies were used: ACSL4 (1:100, Proteintech, 22401-1-AP) and KLF1 (1:100, ThermoFisher, PA5-86441)

### 2.17. Statistical Analysis

All statistical analyses were conducted using GraphPad Prism 8.0 (GraphPad Software Inc., San Diego, CA, USA) and R software (version 3.6.1). Each in vitro experiment was performed in triplicate, and the results are presented as the means  ±  SDs. Differences between the groups were analyzed using Student’s *t*-test, the Mann–Whitney U test, one-way analysis of variance, or the chi-squared test. *p*-values  <  0.05 were considered statistically significant.

## 3. Results

### 3.1. Identification of Differentially Expressed miRNAs, TFs, and Ferroptosis-Related Genes

**Figure 1:**
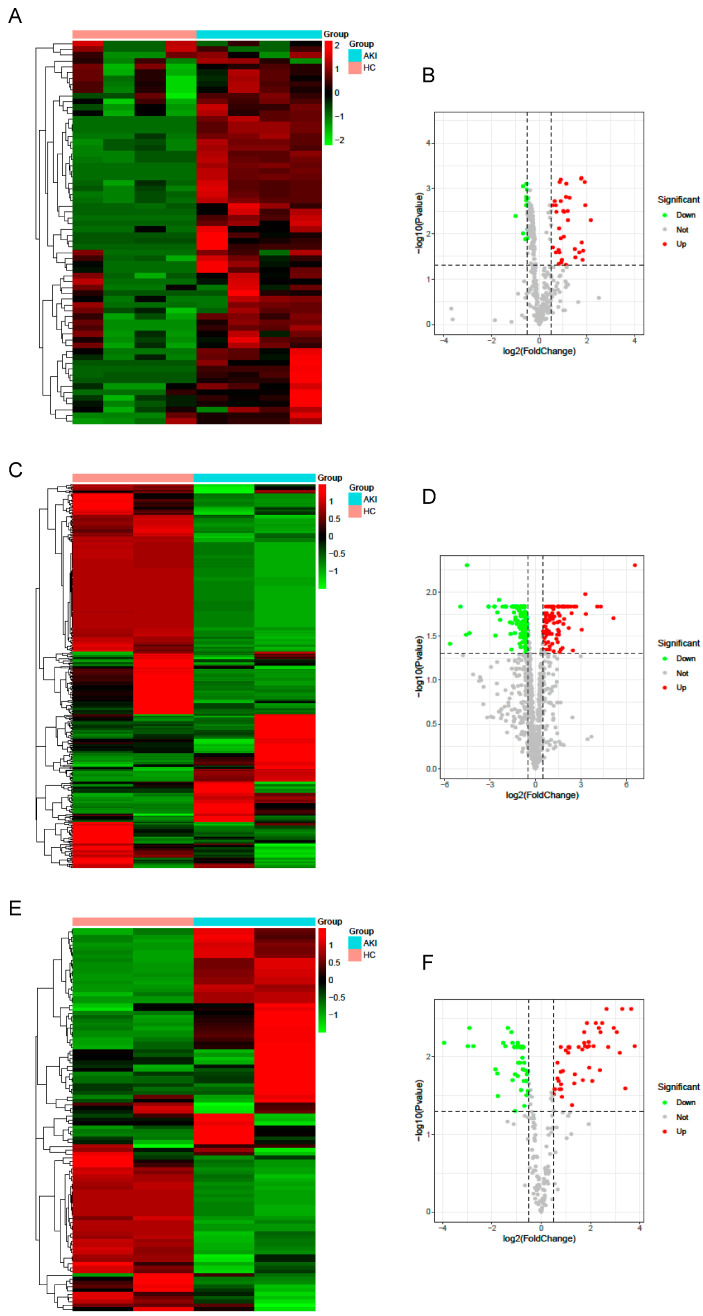
Identifying differentially expressed miRNAs, transcription factors (TFs), and ferroptosis genes. (**A**,**B**) Volcano plot and heatmap showing the differentially expressed miRNAs in GSE172039. (**C**,**D**) Volcano plot and heatmap showing the differentially expressed TFs. (**E**,**F**) Volcano plot and heatmap showing the differentially expressed ferroptosis genes in GSE71647.

To analyze the effects of gene expression on the renal IRI tissues relative to the normal tissues, we conducted limma differential analysis to identify the differentially expressed miRNAs, TFs, and ferroptosis-related genes in the three datasets. Then, we drew a classification heat map and volcano map using the DEGs and data groupings. We identified 66 differentially expressed miRNAs in GSE172039, of which 52 were upregulated and 14 were downregulated (Figure 1A,B). Next, we identified 101 differentially expressed ferroptosis-related genes integrating the GSE71647 dataset and FerrDb database, of which 48 were upregulated and 53 were downregulated (Figure 1C,D). Subsequently, 334 differentially expressed TFs were obtained from GSE71647, of which 92 were upregulated and 242 were downregulated (Figure 1E,F).

### 3.2. Differentially Expressed miRNA–TF–Ferroptosis-Related Gene Network

**Figure 2:**
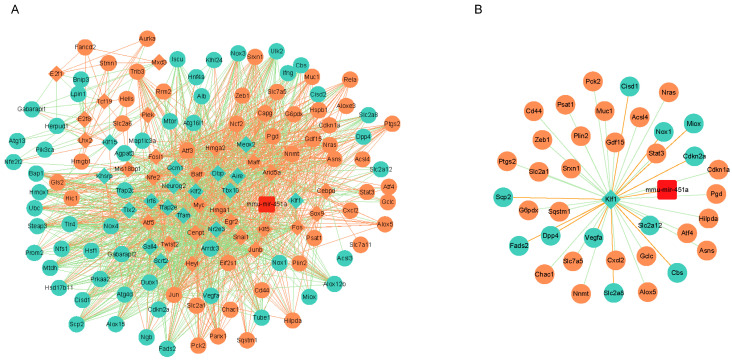
Construction of miRNA–TF–ferroptosis-related genes network. (**A**) The network diagram shows the miRNA–TF–ferroptosis genes. (**B**) The miRNA–TF–ferroptosis genes network of differentially expressed miRNA, TFs, and ferroptosis genes in renal ischemia–reperfusion injury tissue.

First, we constructed a miRNA–TF–ferroptosis-related gene network related to renal IRI, which was visualized in Cytoscape (Figure 2A). Next, we intersected the miRNA, TF, and ferroptosis-related genes from the miRNA–TF–ferroptosis network diagram with the differentially expressed miRNA, TF, and ferroptosis genes, and we obtained the key disease-associated miRNA–TF–ferroptosis axis (Figure 2B). In the network, we noted that miR-451a, significantly enriched in renal IRI tissue, inhibited the expression of KLF1, which regulated the transcription of multiple ferroptosis genes. Specifically, downregulated KLF1 increased the expression of 26 ferroptosis-related differential genes, including ACSL4, ALOX5, ATF4, etc., while reducing the differential expressions of 11 ferroptosis-related genes. Typically, the process of renal IRI is characterized predominantly by lipid peroxidation and the accumulation of reactive oxygen species (ROS). Based on the bioinformatics analysis results, we selected ACSL4, a key enzyme in polyunsaturated fatty acid synthesis, as the target of further investigation.

### 3.3. MiR-451a Upregulated ACSL4 Expression to Promote Ferroptosis During Renal IRI Process

**Figure 3:**
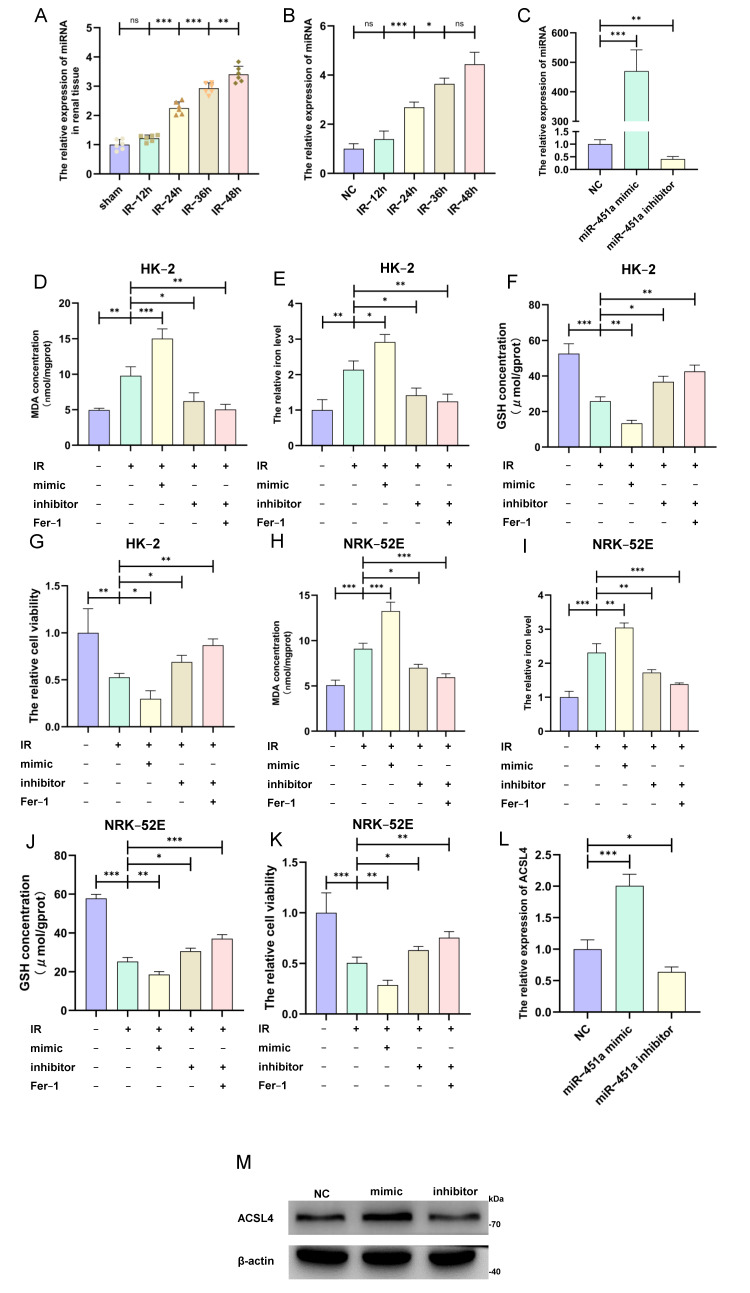
MiR-451a promoted ferroptosis by upregulating ACSL4 expression. (**A**,**B**) MiR-451a was overexpressed in in vivo (n = 6 per group) and in vitro renal ischemia–reperfusion injury models with increasing time. (**C**) miR-451a mimic and inhibitor transfection were effective in HK-2 cell line. (**D**–**G**) The MDA concentration, iron level, GSH concentration, and cell viability were detected after the transfection of the miR-451a mimic and inhibitor in HK-2 cells. (**H**–**K**) The MDA concentration, iron level, GSH concentration, and cell viability were detected after the transfection of the miR-451a mimic and inhibitor in NRK-52E cells. (**L**,**M**) The mRNA and protein expression levels were examined after the transfection of the miR-451a mimic and inhibitor. (All results are from three distinct repetitions. *** *p* < 0.001, ** *p* < 0.01, and * *p* < 0.05 represent significant differences between two groups).

To verify the regulatory function of miR-451a on ferroptosis during the renal IRI process, we established an in situ kidney IRI model and an in vitro IRI model with different durations in the reperfusion stage (12, 24, 36, and 48 h). The qRT-PCR results demonstrate that miR-451a expression was significantly gradually increased with prolonged reperfusion time in the mice renal IRI tissue and HK-2 cells (Figure 3A,B). Subsequently, we transfected the miR-451a mimic and inhibitor into HK-2 cells and verified their expressions (Figure 3C). Next, we demonstrated that miR-451a overexpression remarkably increased the accumulation of MDA and iron while exacerbating the depletion of GSH. Conversely, downregulating miR-451a incurred the opposite results (Figure 3D–F). In addition, the miR-451a mimic obviously aggravated the adverse impact of the IRI process on renal tubular epithelial cells, whereas the inhibitor partially reversed this effect (Figure 3G). Additionally, similar results were observed in the NRK-52E and HK-2 cells (Figure 3H–K). Furthermore, the miR-451a mimic facilitated ferroptosis by promoting the transcriptional and translational expressions of ACSL4; conversely, the inhibitor had an inverse effect on ACSL4 (Figure 3L,M). These findings collectively imply that miR-451a facilitates ferroptosis by inducing ACSL4 expression during the IRI process.

### 3.4. KLF1 Inhibited Ferroptosis and Was Downregulated During Renal IRI Process

**Figure 4:**
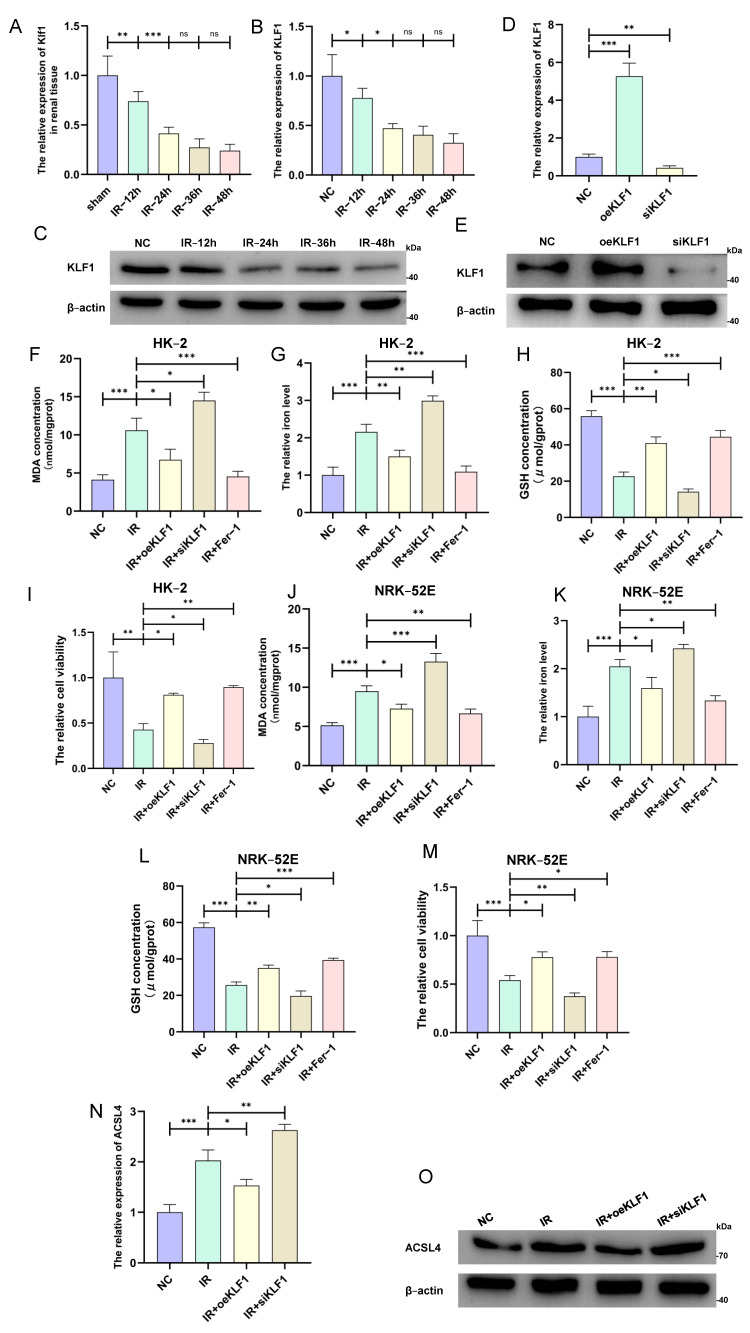
The transcription factor KLF1 is a negative regulator of ACSL4. (**A**–**C**) The mRNA and protein expression levels of KLF1 were decreased in in vivo (n = 6 per group) and in vitro renal ischemia–reperfusion injury models with increasing time. (**D**,**E**) Quantification of transfected KLF1 overexpression and siRNA in HK-2 cells. (**F**–**I**) The MDA concentration, iron level, GSH concentration, and cell viability detected in HK-2 cells. (**J**–**M**) The MDA concentration, iron level, GSH concentration, and cell viability detected in NRK-52E cells. (**N**,**O**) Quantification of the mRNA and protein levels in ACSL4. (All results are from three distinct repetitions. *** *p* < 0.001, ** *p* < 0.01, and * *p* < 0.05 represent significant differences between two groups).

According to the prediction of the network diagram, the transcription factor KLF1 was interestingly inhibited in the IRI renal tissue. The qRT-PCR results demonstrate that the KLF1 expression level was obviously reduced over time in the reperfusion stage, verifying our bioinformatics analysis. The IB results further confirm the downregulation of KLF1 (Figure 4A–C). Then, we transfected the overexpressed plasmid and siRNA and validated their expression levels (Figure 4D,E). Furthermore, in both the HK-2 cells and NRK-52E cells, the MDA concentration and iron levels decreased, whereas the GSH concentration and cell viability increased upon KLF1 overexpression. Using siRNA to silence KLF1 inversely reversed the rescue effect of KLF1 (Figure 4F–M). In addition, KLF1 overexpression similarly lowered the expression of ACSL4 (Figure 4N,O). These results indicate that KLF1 may be a protective factor in preventing IRI renal cells from ferroptosis but is significantly suppressed during the renal IRI process.

### 3.5. MiR-451a–KLF1–ACSL4 Axis Regulated Ferroptosis During Renal IRI Process

**Figure 5:**
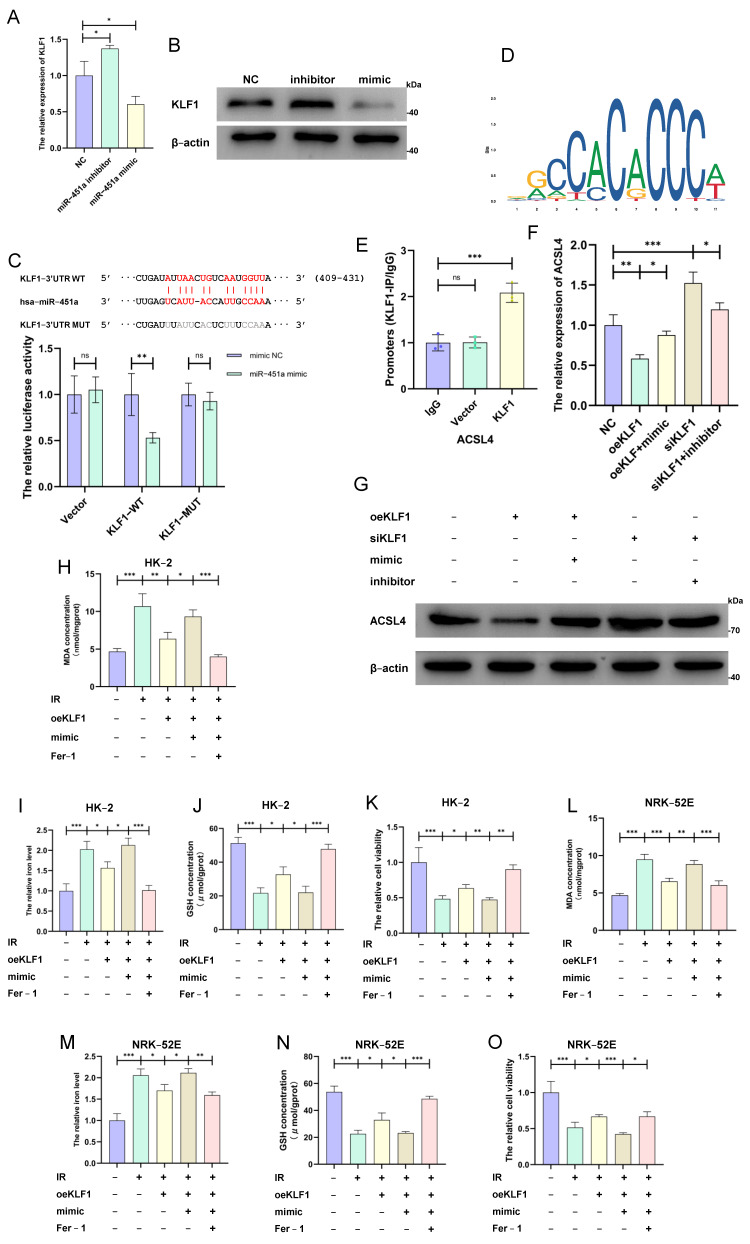
The MiR-451a–KLF1–ACSL4 axis promoted ferroptosis in the in vitro renal ischemia–reperfusion model. (**A**,**B**) The qRT-PCR and IB results demonstrate the KLF1 expression levels after transfection of the miR-451a mimic and inhibitor. (**C**) The luciferase reporter assay results demonstrate that KLF1 was a direct target of miR-451a. (**D**) Online prediction of the interaction sites between KLF1 and ACSL4. (**E**) The levels of ACSL4 promoters binding to KLF1 detected using ChIP–qPCR. (**F**,**G**) The mRNA and protein levels of ACSL4 detected in pretreated HK-2. (**H**–**K**) The MDA concentration, iron level, GSH concentration, and cell viability detected in HK-2 cells. (**L**–**O**) The MDA concentration, iron level, GSH concentration, and cell viability detected in NRK-52E cells. (All results are from three distinct repetitions. *** *p* < 0.001, ** *p* < 0.01, and * *p* < 0.05 represent significant differences between two groups).

To investigate whether KLF1 was the downstream regulatory mechanism of miR-451a according to the miRNA–TF–ferroptosis-related gene network, we first examined the mRNA and protein expression level of KLF1. Surprisingly, the miR-451a mimic and inhibitor down- and upregulated KLF1 mRNA and protein expression in HK-2 cells (Figure 5A,B). Next, we confirmed the direct interaction of miR-451a with KLF1 mRNA 3′-UTR using a luciferase assay. The co-transfection of miR-451a and wild-type KLF1 significantly lowered the luciferase activity, whereas luciferase activity reduction was not seen after the co-transfection of miR-451a and mutant KLF1 (Figure 5C). To verify that ACSL4 was transcriptionally regulated by KLF1 in IRI renal cells, we obtained the binding sites of KLF1 on the ACSL4 promoter region using the JASPAR website (Figure 5D). ChIP–qPCR assays further confirmed that KLF1 was bound to the ACSL4 promoter (Figure 5E). KLF1 overexpression decreased ACSL4 expression, while this change was interestingly reversed by the miR-451a mimic (Figure 5F,G), further validating the regulation of the miR-451a–KLF1–ACSL4 axis. Moreover, the MDA concentration, iron levels, and cell viability were positively correlated with KLF1, and a negative correlation was detected between the GSH concentration and KLF1, whereas the miR-451a mimic and ferroptosis inhibitor in the HK-2 cells and NRK-52E cells neutralized the above relationships (Figure 5H–O). Therefore, the upregulated miR-451a promoted ferroptosis by targeting the KLF1–ACSL4 axis in IRI renal cells.

### 3.6. MiR-451a Promoted Ferroptosis in In Situ Kidney IRI Model

**Figure 6:**
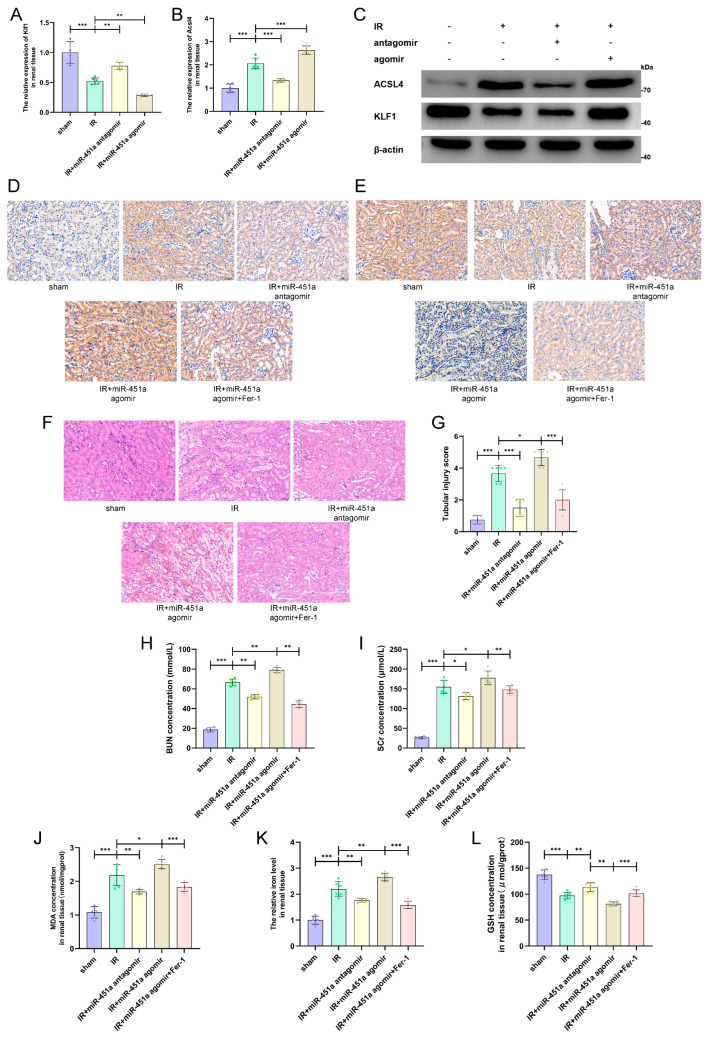
MiR-451a promoted ferroptosis and aggravated renal damage in in vivo renal ischemia–reperfusion injury model. (**A**–**C**) The mRNA and protein levels of KLF1 and ACSL4 were examined in IRI renal tissue. (**D**,**E**) IHC assays detected the expression levels of ACSL4 and KLF1 in renal tissue slices; scale bar: 50 μm. (**F**) HE staining (**left**) and renal pathological score (**right**) of renal tissue slice; scale bar: 50 μm. (**G**,**H**) The SCr and BUN concentrations of mice after various treatments. (**I**–**L**) The MDA concentration, iron level, and GSH concentration were detected in renal tissue. (All results are from six distinct repetitions. *** *p* < 0.001, ** *p* < 0.01, and * *p* < 0.05 represent significant differences between two groups).

Finally, we established a mouse in situ kidney IRI model combined with various treatments with miR-451a agomir, antagomir, and Fer-1. We first detected that the KLF1 expression levels were downregulated by miR-451a agomir and upregulated by antagomir, while the opposite occurred on ACSL4 (Figure 6A–C). As expected, the IHC results further confirm the qRT-PCR and IB results (Figure 6D,E). We further detected that miR-451a sharply aggravated morphological and functional graft injury, as indicated by the H&E staining results (Figure 6F,G) and the SCr and BUN concentrations (Figure 6H,I). Importantly, the results of the regulatory effects of miR-451a on ferroptosis in renal tissues are broadly in line with the morphological and functional changes (Figure 6J–L).

## 4. Discussion

Renal IRI is a significant clinical condition characterized by a temporary reduction in blood flow to the kidneys followed by the restoration of perfusion [1]. This process initiates a complex cascade of cellular and molecular events, resulting in acute kidney injury (AKI), delayed graft function (DGF), and other pathological processes [25,26]. Renal IRI is a common complication in various clinical scenarios, including renal transplantation, cardiovascular surgery, and severe trauma, often leading to significant morbidity and mortality [3,4]. A comprehensive understanding of the underlying mechanisms is essential for the development of therapeutic strategies to mitigate renal IRI. Here, we report for the first time that miR-451a is a promising novel diagnostic biomarker and therapeutic target for renal IRI.

Accumulating evidence has underscored the role of microRNAs in renal IRI [27]. For instance, the targeted deletion of Dicer in the proximal tubular epithelium confers protection against renal IRI, and this protective effect is associated with changes in the expressions of multiple microRNAs [28]. Additionally, increased expression of miR-21 has been observed in proliferating tubular epithelial cells, while the knockdown of miR-21 in these cells resulted in heightened apoptosis [29]. Another study reported that miR-127 was induced during both ischemia and reperfusion in in vivo and in vitro models, mediated by hypoxia-inducible factor-1alpha (HIF-1α) stabilization, and was involved in maintaining cell–matrix and cell–cell adhesion [30]. Here, we reveal that miR-451a was significantly enriched in IRI renal tubular epithelial cells, further promoting ferroptosis and exacerbating renal damage.

Furthermore, we successfully predicted and validated that KLF1 is a downstream target gene of miR-451a. KLF1 is a transcriptional regulator that plays a critical role in the control of gene expression. Previous studies have shown that KLF1 predominantly participates in maintaining the proper function and development of the erythroid lineage [31,32]. However, the role of KLF1 in the context of renal IRI remains poorly understood. In our study, we predicted and successfully verified that KLF1 acts as a negative regulator of ACSL4. Further results demonstrate that KLF1 expression was significantly reduced by upregulated miR-451a, leading to a diminished suppression of ACSL4. This modulation resulted in the facilitation of ferroptosis in both the in vivo and in vitro models of renal IRI.

Ferroptosis is a novel type of cell death characterized by the accumulation of iron and lipid peroxidation. The iron-dependent accumulation of lipid ROS is integral to the process of ferroptosis. ACSL4 is involved in the biosynthesis and remodeling of phosphatidylethanolamine (PE) and activates polyunsaturated fatty acids (PUFAs), which are essential elements for ferroptosis [6]. Gao et al. reported that the inhibition of glutamine metabolism can reverse heart IRI by influencing ferroptosis levels [33]. Additionally, Li et al. demonstrated that lncRNA-WAS-AS1, loaded in small extracellular vesicles, could induce ferroptosis in neighboring cells, ultimately exacerbating renal damage [34]. Our analysis demonstrated that ferroptosis levels were activated and promoted by the miR-451a–KLF1–ACSL4 axis.

In conclusion, our study revealed that miR-451a was highly overexpressed in the context of renal ischemia–reperfusion injury (IRI). This overexpression of miR-451a led to the negative regulation of its downstream target, KLF1, a key transcriptional regulator. The downregulation of KLF1 resulted in reduced suppression of ACSL4 expression, an enzyme associated with lipid metabolism and ferroptosis. Consequently, the elevated levels of ACSL4 promoted ferroptosis, thereby exacerbating renal damage during IRI. These findings underscore the significant role of miR-451a in the pathophysiology of renal IRI and suggest that targeting miR-451a could be a potential therapeutic strategy for mitigating IRI-induced renal injury, which is particularly relevant in clinical settings such as kidney transplantation. However, our study’s limitations include reliance on animal and in vitro models (which may not fully mimic human IRI), the absence of in vivo therapeutic interventions, potential off-target effects of miR-451a modulation, and an insufficient clinical sample correlation to validate the findings.

## 5. Conclusions

Our study found that miR-451a was significantly upregulated in renal IRI tissue and cells, which reduced the expression of the transcription factor KLF1 and ultimately promoted ferroptosis during the renal IRI process. These findings may shed new light on the underlying mechanisms and provide a novel therapeutic target for renal IRI.

## Data Availability

The original contributions presented in this study are included in the article. Further inquiries can be directed to the corresponding author.
